# Supplementary material for: PNPLA1 is a transacylase essential for the generation of the skin barrier lipid ω-O-acylceramide
Source: Nat Commun. 2017 Mar 1;8:14610. doi: 10.1038/ncomms14610 (PMC5337975; doi:10.1038/ncomms14610)
Supplement: Supplementary Information — Supplementary Figures and Supplementary Tables. [file ncomms14610-s1.pdf]

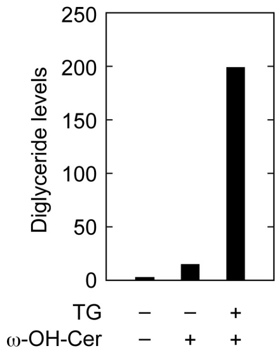

Supplementary Figure 1 | Diglyceride is increased by a PNPLA1-dependent transacylation reaction. Proteoliposomes containing PNPLA1 were incubated for 1 h at 37 °C in the presence or absence of TG and C30:0 ω-hydroxyceramide (ω-OH-Cer), as indicated. Lipids were extracted, and the diglyceride containing linoleic acid was quantified by LC-MS/MS analysis in MRM mode selecting the *m/z* 634.5 at Q1 and the *m/z* 337.3 at Q3. Values represent the amount of diglyceride relative to the control (without TG or C30:0 ω-hydroxyceramide).

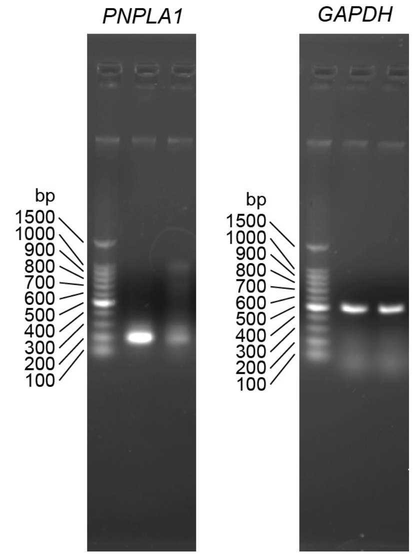

Supplementary Figure 2 | Uncropped scanned photographs. Uncropped scanned photographs of the electrocataphoresis gels in Fig. 2a are presented.

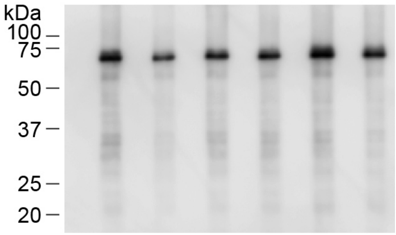

Supplementary Figure 3 | An uncropped scan of the blot. An uncropped scan of the blot in Fig. 5a is presented.

Supplementary Table 1 | Primers used in this study

Primer	Sequence
ABHD5-F	5'-GGATCCATGGCGGCGGAGGAGGAGGAGGTGG-3'
ABHD5-R	5'-TCAGTCCACAGTGTCGCAGATCTCC-3'
LIPN-F	5'-AGATCTATGATGTGGCTGCTTTTAACAACAAC-3'
LIPN-R	5'-TTAGGAATATGCCTTCATTAAAGC-3'
PNPLA1-F	5'-AGATCTATGGAAGAACAGGTGTTCAAGGGGG-3'
PNPLA1-R	5'-TCACTGCACTTTGCTGCTTGGTTTTTTGG-3'
PLA2G15-F	5'-AGATCTATGGGCCTCCACCTCCGCCCCTACC-3'
PLA2G15-R	5'-TCAGGGCCCAAGGAGCACACGTTTC-3'
LCAT-F	5'-AGATCTATGGGGCCGCCCGGCTCCCCATGGC-3'
LCAT-R	5'-TTATTCAGGAGGCGGGGGCTCTGGG-3'
DGAT2-F	5'-AGATCTATGAAGACCCTCATAGCCGCCTACTC-3'
DGAT2-R	5'-TCAGTTCACCTCCAGGACCTCAGTC-3'
shPNPLA1-F	5'-ACCTCGGGTGAATAAGGTCTTCAATTCAAGAGATTGAAG ACCTTATTCACCCTT-3'
shPNPLA1-R	5'-CAAAAAGGGTGAATAAGGTCTTCAATTCAAGAGATTGA AGACCTTATTCACCCG-3'
PNPLA1-F2	5'-AACTAGGCCAAGAACAGCCC-3'
PNPLA1-R2	5'-CCGAATGTCTTGGGAAGCCT-3'
GAPDH-F	5'-CTGTTCGACAGTCAGCCGCATCTTC-3'
GAPDH-R	5'-CCGTTGACTCCGACCTTCACCTTCC-3'
PNPLA1 A34T-F	5'-CAGGTGCTGTGATCGTCGCCCTGGCCATCTG-3'
PNPLA1 A34T-R	5'-CAGATGGCCAGGGCGACGATCACAGCACCTG-3'
PNPLA1 A59V-F	5'-GCGGGGGCTGTGGACACCCTGCGGGACCTGG-3'
PNPLA1 A59V-R	5'-CCAGGTCCCGCAGGGTGTCCACAGCCCCCGC-3'
PNPLA1 E131X-F	5'-CGCTTAACGGACGGGTAGAATGTGGTGGTTTC-3'
PNPLA1 E131X-R	5'-GAAACCACCACATTCTACCCGTCCGTTAAGCG-3'

Supplementary Table 2 | Selected *m/z* values and collision energies for acylceramide species in MS/MS analysis

**Acylceramide species**	**Precursor ion (Q1)**	**Product ion (Q3)**	**Collision energy (V)**
d18:1/C26:1/C18:2	936.8, 954.8	264.2	35
d18:1/C26:0/C18:2	938.8, 956.8	264.2	35
d18:1/C28:1/C18:2	964.8, 982.8	264.2	35
d18:1/C28:0/C18:2	966.8, 984.8	264.2	35
d18:1/C30:1/C18:2	992.8, 1010.8	264.2	40
d18:1/C30:0/C18:2	994.8, 1012.8	264.2	40
d18:1/C32:1/C18:2	1020.8, 1038.8	264.2	40
d18:1/C32:0/C18:2	1022.8, 1040.8	264.2	40
d18:1/C34:1/C18:2	1048.8, 1066.8	264.2	40
d18:1/C34:0/C18:2	1050.8, 1068.8	264.2	40
d18:1/C36:1/C18:2	1076.8, 1094.8	264.2	45
d18:1/C36:0/C18:2	1078.8, 1096.8	264.2	45

Supplementary Table 3 | Selected *m/z* values for TG species in MS/MS analysis

TG species	Precursor ion (Q1)	Product ion (Q3)
C18:2/C30:1	818.8	521.5
C18:2/C30:0	820.8	523.5
C18:2/C32:1	846.8	549.5
C18:2/C32:0	848.8	551.5
C18:2/C34:2	872.8	575.5
C18:2/C34:1	874.8	577.5
C18:2/C34:0	876.8	579.5
C18:2/C36:3	898.8	601.5
C18:2/C36:2	900.8	603.5
C18:2/C36:1	902.8	605.5
C18:2/C36:0	904.8	607.5
